# Novel cationic aryl bithiophene/terthiophene derivatives as corrosion inhibitors by chemical, electrochemical and surface investigations

**DOI:** 10.1038/s41598-022-06863-8

**Published:** 2022-02-24

**Authors:** Mohamed A. Ismail, Mahmoud M. Shaban, Ehab Abdel-Latif, Fatma H. Abdelhamed, Mohamed A. Migahed, Mahmoud N. El-Haddad, Ashraf S. Abousalem

**Affiliations:** 1 Department of Chemistry, Faculty of Science, Mansoura University, Mansoura, 35516 Egypt; 2 Egyptian Petroleum Research Institute (EPRI), Nasr City, 11727 Cairo Egypt; 3 Operation Department, Quality Control Laboratory, Jotun, Egypt

**Keywords:** Corrosion, Electrochemistry

## Abstract

Two novel bithienyl fluorobenzamidine derivatives namely, 4-([2,2′:5′,2′′-terthiophen]-5-yl)-2-fluorobenzamidine hydrochloride salt (MA-1615), 5′-(4-amidino-3-fluorophenyl)-[2,2′-bithiophene]-5-carboxamidine dihydrochloride salt (MA-1740) were synthesized, characterized and their corrosion inhibition properties were evaluated by electrochemical methods for carbon steel (C-steel) in 1 M HCl. Experimental investigations revealed that the inhibition effectiveness of the investigated inhibitors (INHs) by the Tafel polarization method followed the order: MA-1740 (96.9%) > MA-1615 (95.6%), demonstrating higher efficiency than inhibitors of similar structure reported in the literature. The investigated bithiophene derivatives exhibit mixed-type corrosion inhibition characteristics by blocking the active sites on the surface of C-steel. EIS study revealed that the INHs behave as interface-type corrosion inhibitors. UV–Visible spectrometric measurements confirmed a complex formation between the Fe^2+^ cation released during the corrosion reactions and inhibitor molecules.

## Introduction

Corrosion is commonly defined as the degradation of materials as a result of interaction with the surrounding environment. It occurs to metallic materials when exposed to corrosive environments, causing destructive industrial problems that result in a huge economic loss for many countries around the world. Not only does corrosion cause huge material loss, but also compromise human safety^1,2^. Despite the large number of ongoing research projects that aimed to design new corrosion resistant materials to cater for different types of industrial applications, C-steel is still the black horse among other metals due to its versatility, cost effectiveness, and excellent mechanical properties. However, one main drawback of using it in industries is that it easily corrodes when exposed to aggressive acids such as hydrochloric acid, which is widely used in oil and gas operations such as pickling, cleaning, and oil-well acidizing^3^. Nowadays, investigating the inhibitory performance of C-steel corrosion in such acid medium has received a considerable attention in academia and across the industrial domain. Over the last three decades, there have been some studies devoted to the development of efficient and cost-effective corrosion inhibitors. One of the most promising class of inhibitors are those organic compounds with hetero-atoms such as nitrogen, oxygen, sulfur, and phosphorus, which have the ability to adsorb on a metallic surface, and thereby protecting the surface from corrosion attack^4^. Bi- and oligochalcophenes play an important role as advanced materials, which have been applied into numerous fields and applications. These new class of organic compounds can be used as organic semiconductors and radioactive materials due to their luminescent properties^5^. Thiophene-containing cationic compounds are important synthetic precursor for biologically active molecules^6–8^. These types of compounds have significant use in solvatochromic, photosensitizing, and photovoltaic cells applications^9–12^ The idea depends on the presence of donor–(π-spacer)–acceptor in conjugation with each other consequently^13,14^. These compounds bear cyano group/or cationic amidine group which act as electron withdrawing acceptor, and electron donating group representing in thiophene ring. Owing to growing concerns on the toxic effects of such corrosion inhibitors, ongoing studies are conducted to develop new compounds which have safe impact on environment. The corrosion inhibition performance of bichalcophene compounds have been previously investigated in literature for C-steel alloys. The maximum %IE results EIS corrosion measurements are summarized in Table 1. In the present investigation, we continue our work on studying new terthiophene and bithiophenes derivatives owing to its non-toxicity profile meriting corrosion efficiency of these compounds reported in our previous work. Terthiophene inhibitors are naturally occurring compounds^15–17^. The readability and accessibility of certain compounds that occur naturally in our local environment is a major advantage in harnessing their potentials as potential eco-friendly corrosion inhibitors. We aim in this study to advance our work by introducing two novel compounds and bench their corrosion inhibition potency against other bichalcophene compounds that previously reported in literature. This study encompasses synthesis work, experimental studies, surface analysis.

**Table 1:** Corrosion inhibition efficiencies by EIS of previously studied bichalcophene derivatives in literature.

Inhibitor	Material	Media	%IE	Year	References
4-(2,2′-Bithiophene-5-yl) benzamidine	C-steel	1 M HCl	82.6	2017	^18^
6-(2,2′-Bithiophene-5-yl) nicotinamidine			87.7		
4-(2,2′-Bifuran-5-yl)benzamidine	C-steel	1 M HCl	66.1	2017	^19^
6-(2,2′-Bifuran-5-yl)nicotinamidine			64.5		
6-[5-(Thiophen-2-yl)furan-2-yl]nicotinamidine			78.7		
[2,2′:5′,2′′-Terthiophene]-5-amidine	C-steel	1 M HCl	86.6	2019	^20^
5′-Phenyl-2,2′-bithiophene-5-amidine			77.9		
5′-(4-Methoxyphenyl)-2,2′-bithiophene-5-amidine			80.8		
5′-(3,5-Dimethoxyphenyl)-2,2′-bithiophene-5-amidine			83.6		
4-([2,2′-Bithiophen]-5-yl)-2-fluorobenzamidine	C-steel	1 M HCl	86.4	2019	^3^
4-([2,2′:5′,2′′-Terthiophen]-5-yl)-2-fluorobenzamidine hydrochloride salt (MA-1615)	C-steel	1 M HCl	93.1	2021	Our work
5′-(4-Amidino-3-fluorophenyl)-[2,2′-bithiophene]-5-carboxamidine dihydrochloride salt (MA-1740)			94.6		

## Materials and methods

The structure and molecular formula of studied cationic aryl bithiophene/terthiophene derivatives MA-1615, and MA-1740 are given in Table 2. The full details on the synthesis process and characterization (For ^1^H-NMR, ^19^F-NMR, and Mass spectra of inhibitors see the Electronic Supplementary Material) are in the following part. The investigated inhibitors contain the same counter ion Cl^−^, so we assume that this counter ion has little effect in the prevailing acid environment of 1.0 M HCl compared to the cationic part.

**Table 2:** Molecular structures, formulas, weights of bithiophene derivatives.

Inhibitor code	Molecular structures/chemical names	Mol. formulas (F. wt)
MA-1615		C_19_H_13_FN_2_S_3_-1.0HCl (420.97)
MA-1740		C_16_H_13_FN_4_S_2_-2.0HCl (417.35)

### Methodology for preparation of inhibitors

#### Monocationic terthiophene derivative 4

##### 4-(5′-Bromo-[2,2′-bithiophen]-5-yl)-2-fluorobenzonitrile (2)

Bromobithiophene derivative **2** was prepared starting from 4-([2,2′-bithiophen]-5-yl)-2-fluorobenzonitrile **(1)**^21^, on treatment with NBS in DMF adopting the reported methodology^21^. Bromobithiophene compound **2** was obtained as a yellow solid in 87% yield, m.p. 170–171 °C (EtOH). R_f_ = 0.72, petroleum ether-EtOAc (8:2). IR (KBr) ν/cm^−1^; 3088 (CH, stretch), 2230 (CN, stretch), 1615, 1555, 1520 (C=C, stretch). ^1^H-NMR (DMSO-*d*_6_); δ 7.24–7.27 (m, 2H), 7.42 (d, J = 4.0 Hz, 1H, thiophene-H), 7.66 (d, J = 8.4 Hz, 1H), 7.81 (d, J = 4.0 Hz, 1H, thiophene-H), 7.87–7.94 ppm (m, 2H). MS (EI) m/e (rel.int.); 363, 365 (M^+^, 94, 100: bromine isotopes). Anal. Calcd. for: C_15_H_7_BrFNS_2_ (364.25): C, 49.46; H, 1.94; N, 3.85 Found: C, 49.23; H, 2.05; N, 3.77%.

##### 4-([2,2′:5′,2′′-Terthiophen]-5-yl)-2-fluorobenzonitrile (3)

Terthienyl compound **3** was prepared adopting a Stille coupling conditions between 4-(5′-bromo-[2,2′-bithiophen]-5-yl)-2-fluorobenzonitrile (**2**), and 2-tributyltin thiophene adopting the reported methodology^21^. Compound **3** was obtained as an orange solid in 54% yield, m.p. 211–212 °C (DMF/EtOH). R_f_ = 0.63, petroleum ether-EtOAc (8:2). IR (KBr) ν/cm^−1^; 3067 (CH, stretch), 2228 (CN, stretch), 1612, 1556, 1513 (C=C, stretch). ^1^H-NMR (DMSO-*d*_6_); δ 7.10–7.12 (m, 1H) 7.32 (d, J = 4.0 Hz, 1H, thiophene-H), 7.37 (m, 1H), 7.39 (d, J = 4.0 Hz, 1H, thiophene-H), 7.46 (d, J = 4.0 Hz, 1H, thiophene-H), 7.55–7.57 (m, 1H), 7.67 (dd, J = 8.0, 1.5 Hz, 1H), 7.83 (d, J = 4.0 Hz, 1H, thiophene-H), 7.88–7.95 ppm (m, 2H). MS (EI) m/e (rel.int.); 367 (M^+^, 100). Anal. Calcd. for C_19_H_10_FNS_3_ (367.48): C, 62.10; H, 2.74; N, 3.81 Found: C, 61.93; H, 2.92; N, 3.76%.

##### 4-([2,2′:5′,2′′-Terthiophen]-5-yl)-2-fluorobenzamidine hydrochloride salt (4)

Terthienylbenzamidine compound **4** was prepared by treatment of terthienyl-fluorobenzonitrile compound **3** with lithium bis-trimethylsilylamide adopting reported methodology^21^. Compound **4** was obtained as a reddish-brown solid in 63% yield, m.p. 292–293 °C. IR (KBr) ν/cm^−1^; 3389 (NH, stretch), 3063 (CH, stretch), 1655, 1618, 1525 (C=N, C=C, stretch, NH, bending). ^1^H-NMR (DMSO-*d*_6_); δ 7.09–7.12 (m, 1H), 7.30–7.46 (m, 4H), 7.56 (d, J = 4.5 Hz, 1H, thiophene-H), 7.64–7.72 (m, 2H), 7.80–7.86 (m, 2H), 9.38 (s, 2H, NH_2_; D_2_O exchangeable), 9.49 ppm (s, 2H, ^+^NH_2_; D_2_O exchangeable). ^19^F-NMR (DMSO-*d*_6_); − δ112.88 ppm (using TFA as external standard). MS (EI) m/e (rel.int.); 384 (M^+^, 99), 385 (M^+^ + 1, 100), 367 (M^+^-NH_3_, 72). Anal. Calcd. for C_19_H_13_FN_2_S_3_-1.0HCl (420.97): C, 54.21; H, 3.35; N, 6.65 Found: C, 54.37; H, 3.42; N, 6.53%.

#### Dicationic bithiophene-5-carboxamidine derivative 6

##### 5′-(4-Cyano-3-fluorophenyl)-[2,2′-bithiophene]-5-carbonitrile (5)

A mixture of 4-(5′-bromo-[2,2′-bithiophen]-5-yl)-2-fluorobenzonitrile (**2**) (910 mg, 2.50 mmol) and Cu(I)CN (270 mg, 3 mmol) in dry DMF (25 mL) was refluxed at 120–130 °C for 48 h. The reaction mixture was poured onto water/ammonia and extracted with ethyl acetate. The extract was washed with water and brine, dried over Na_2_SO_4_, then recrystallized from ethanol/EtOAc to afford the dicarbonitrile compound **5** as a yellow solid in 52% yield, m.p. 221–222.5 °C. R_f_ = 0.62, petroleum ether-EtOAc (8:2). IR (KBr) ν/cm^−1^; 3090 (CH, stretch), 2212 (2CN, stretch), 1614, 1551, 1489 (C=C, stretch). ^1^H-NMR (DMSO-*d*_6_); δ 7.57 (d, J = 4.0 Hz, 1H, thiophene-H), 7.67 (d, J = 4.0 Hz, 1H, thiophene-H), 7.71 (dd, J = 8.5, 2.0 Hz, 1H, Ar–H of fluorobenzonitrile ring), 7.89 (d, J = 4.0 Hz, 1H, thiophene-H), 7.93–7.96 (m, 2H, Ar–H of fluorobenzonitrile ring), 7.98 ppm (d, J = 4.0 Hz, 1H, thiophene-H). MS (EI) m/e (rel.int.); 310 (M^+^, 100). Anal. Calcd. for: C_16_H_7_FN_2_S_2_ (310.37): C, 61.92; H, 2.27; N, 9.03 Found: C, 62.05; H, 2.38; N, 8.83%.

##### 5′-(4-Amidino-3-fluorophenyl)-[2,2′-bithiophene]-5-carboxamidine dihydrochloride salt (6)

Bithiophene diamidine compound **6** was prepared adopting the same conditions used for preparation of carboxamidine compound **4**. Compound **6** was obtained in 69% yield as a yellowish-brown solid, m.p. 292–294 °C. IR (KBr) ν/cm^−1^; 3342, 3187 (NH, stretch), 3087 (CH, stretch), 1663, 1619, 1523 (C = N, C = C stretch & NH bending). ^1^H-NMR (DMSO-*d*_6_); δ 7.65 (d, J = 4.0 Hz, 1H, thiophene-H), 7.66 (d, J = 4.0 Hz, 1H, thiophene-H), 7.73–7.75 (m, 2H, Ar–H of fluorobenzamidine ring), 7.89 (d, J = 4.0 Hz, 1H, thiophene-H), 8.13 (d, J = 4.0 Hz, 1H, thiophene-H), 7.91 (d, J = 8.5 Hz, 1H, Ar–H of fluorobenzamidine ring), 9.22 (s, 2H, NH_2_; D_2_O exchangeable), 9.45 (s, 2H, NH_2_; D_2_O exchangeable), 9.51 (s, 2H, ^+^NH_2_; D_2_O exchangeable), 9.54 ppm (s, 2H, ^+^NH_2_; D_2_O exchangeable). ^19^F-NMR (DMSO-*d*_6_); − δ112.76 ppm (using TFA as external standard). MS (EI) m/e (rel.int.); 344 (M^+^, 56), 327 (M^+^-NH_3_, 100), 310 (M^+^-2NH_3_, 49). Anal. Calcd. for C_16_H_13_FN_4_S_2_-2.0HCl (417.35): C, 46.05; H, 3.62; N, 13.42 Found: C, 45.78; H, 3.78; N, 13.17%.

### Corrosion measurements

#### Electrochemical measurements

Corrosion tests were conducted on API 5L X70 grade C-steel specimen with the following elemental structure (wt. %): C, 0.026; Mn, 1.51; Si, 0.10; S, 0.02; N, 0.27; Ni, 0.16; Al, 0.35; Cr, 0.27; Cu, 0.28; Nb, 0.93; Ti, 0.11; and remainder Fe. The corrosive electrolyte of 1 M HCl was freshly prepared by dilution of analytical grade HCl with a concentration of 37% using bi-distilled water. The concentration of MA-1615 and MA-1740 inhibitors in corrosive solution ranged from 1 (1 × 10^6^ M) to 500 µM (5 × 10^4^ M). Volta lab 80 (Tacussel-Radiometer PGZ-402) was used for electrochemical tests {Potentiodynamic polarization measurements (PP) and Electrochemical impedance spectroscopy (EIS)}. Electrochemical tests were executed by the typical 250 ml sealed electrochemical glass cell containing a finely cut API 5L X70 grade C-steel as working specimen, with an exposed surface area for 1 cm^2^^22^. Platinum metallic sheet was mounted as the auxiliary electrode, and saturated calomel electrode (SCE) was utilized as the reference electrode. The surface of the working electrode was polished to mirror finish with various grades of emery papers (from 320 to 2000), degreased by acetone, cleaned by demineralized water and finally dried at room temperature before starting the tests. The reference electrode was placed close to the working electrode surface using Luggin–Haper capillary to nullify the iR potential drop between both the electrodes ends. EIS measurements were conducted via AC signal (10 mV) peak to peak, in potentiostatic mode at open-circuit steady state potentials, in the frequency extent between 100 kHz and 20 mHz^23^. Potentiodynamic polarization (PP) tests were executed using the scan rate at 1 mV s^−1^ and sweeping the electrode potential ± 300 mV relative to the steady state potential^24^. To ensure the consistency and accuracy of the results, all tests had been repeated three times.

#### Weight loss measurements

Tests were performed on petroleum pipeline alloy API 5L X70 carbon steel with density, D = 7.86 g cm^−3^ and with the same chemical composition of the electrode material. The carbon steel coupons were fabricated with dimension 3 × 2 × 0.2 cm. The surface of tested coupons was abraded using various grades of emery papers (from 320 to 2000), degreased with acetone, cleaned by demineralized water and finally dried at room temperature before starting the weight loss experiments. After weighing accurately, the specimens were immersed in 250 ml beaker, which contained 150 ml aggressive solution with and without addition of different concentrations of the tested inhibitors. After the required immersion time (48 h), the test specimens were removed, washed with double distilled water, dried by a jet of air and finally weighed. The surface coverage area (θ) for the different concentrations of the investigated inhibitors in 1 M HCl “aggressive solution” and the inhibition efficiency from weight loss, η_w_ (%) were calculated by the following Eq. (1):

1 $${\eta }_{w}\left(\%\right)=\theta \times 100=\left(\frac{\Delta W-{\Delta W}_{i}}{\Delta W}\right)\times 100,$$

where, ΔW and ΔW_i_ are the weight loss per unit area in absence and presence of additive, respectively. The rate of corrosion (K) in (mg cm^−2^ h^−1^) was calculated by the following Eq. (2):

2 $$K=\frac{\Delta W}{St},$$

where, ΔW is the average weight loss of three parallel carbon steel sheets in mg, S is the total area of the specimen in cm^2^ and t is the immersion time in hour.

#### UV–visible spectra measurement

UV–visible absorption spectrometric method was carried out for (500 µM) of inhibitor solution, and the corrosive solution containing (500 µM) of investigated inhibitor after immersion of C-steel sample at 30 °C for 28 h. The spectra were measured using a PG instruments T80+ spectrometer.

### Morphology analysis

Morphological investigation was carried out by Nanosurf Flex AFM for API X70 type C-steel samples, with dimensions 10 × 10 × 2 mm. The API 5L X70 steel surface was pretreated in the identical way of the working electrode and immersed for 24 h in 1 M HCl solution without and with 0.001 M of MA-1740 at 298 ± 1 K. Samples were collected, and then dried for analysis with AFM^25^.

## Results and discussion

### Synthesis and structure elucidation of inhibitors

#### Monocationic terthiophene derivative 4

**Figure 1:**
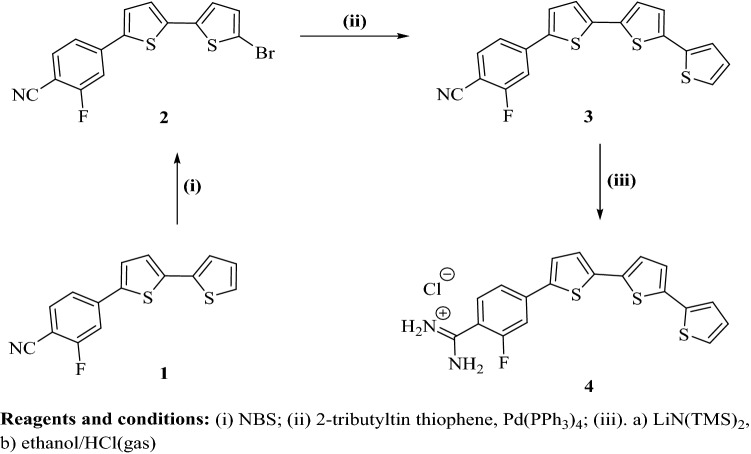
Synthesis scheme for the new α-terthienylbenzamidine derivative **4**.

The preparation of the new terthienylbenzamidine derivative **4** (Fig. 1) begins with bromination reaction of bithiophene derivative **1**^21^ with NBS to afford bromo bithiophene compounds **2** followed by a Stille coupling reaction with 2-tributyltin thiophene to furnish the terthienylbenzonitrile **3**. Benzonitrile derivative **3** was allowed to react with lithium bis-trimethylsilylamide, followed by hydrolysis with hydrogen chloride. The resultant precipitate was neutralized with 1 N NaOH to furnish the corresponding free base of terthienylbenzamidine **4**. The terthienylbenzamidine hydrochloride salt **4** was prepared by treatment of the proper free base of monoamidine with hydrogen chloride in ethanol.

Structure of the newly synthesized terthienylbenzonitrile derivative **3** was assigned based on its elemental and spectral analyses. IR spectra of the terthienylbenzonitrile **3** indicated the presence of nitrile group with stretching vibrations at 2228 cm^−1^. ^1^H-NMR spectrum of the fluorobenzonitrile derivative **3** displayed seven signal integrated for seven protons of terthienyl moiety and two signals integrated for three protons of 1,3,4-trisubstituted benzene ring. Mass spectrum of compound **3** gave a molecular ion peak m/z at 367 (M^+^) as a base peak. Structure of the newly synthesized terthienybenzamidine derivative **4** was assigned based on its elemental and spectral analyses. IR spectra of compound **4** indicated the disappearance of nitrile group and displayed new peaks corresponding for N–H stretching vibrations at ν′ 3460, 3389 cm^−1^. ^1^H-NMR spectrum of the monocationic compound **4** gave two singlet signals at δ 9.38 (2H, NH_2_) and 9.49 (2H, ^+^NH_2_) of the cationic amidine group, plus five signals integrated for ten protons of terthienyl moiety and 1,3,4-trisubstituted benzene ring. ^19^F-NMR displayed a singlet signal at − δ112.88 ppm (using TFA as external standard) of fluorine atom. Mass spectrum of terthienyl fluorobenzamidine derivative **4** gave a m/z peak at 384 of its molecular ion peaks (M^+^), and m/z peak at 385 (M^+^ + 1) as a base peak, along with a fragment with m/z peak at 367 due to loss of a molecule of ammonia.

#### Dicationic bithiophene-5-carboxamidine derivative 6

**Figure 2:**
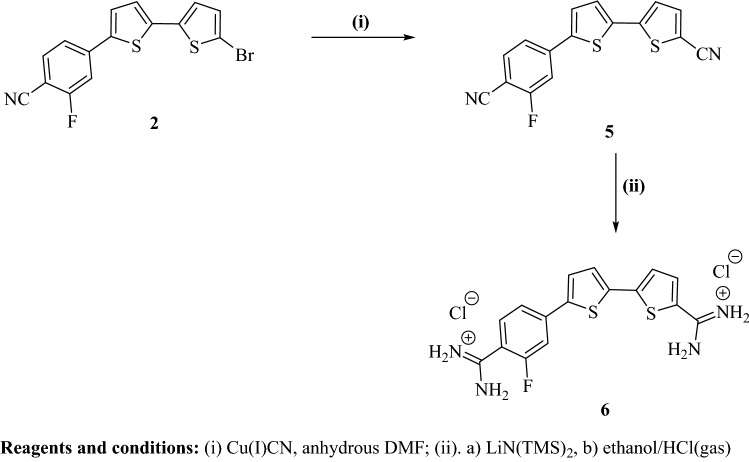
Synthesis scheme for dicationic bithiophene-5-carboxamidine derivative **6**.

The preparation of the new bithiophene diamidine **6** (Fig. 2) begins with cyanation of 4-(5′-bromo-[2,2′-bithiophen]-5-yl)-2-fluorobenzonitrile (**2**) with Cu(1)CN in DMF at 120–130 °C to afford the dicarbonitrile derivative **5**. Dicarbonitrile derivative **5** was allowed to react with LiN(TMS)_2_, followed by hydrolysis with hydrogen chloride. The resultant precipitate was neutralized with 1 N NaOH to furnish the corresponding free base of diamidine **6**. The diamidine hydrochloride salt **6** was prepared by treatment of its free base of diamidine with hydrogen chloride in ethanol.

**Figure 3:**
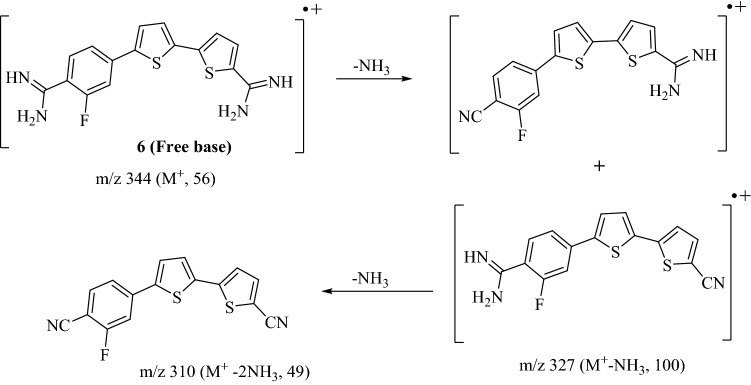
Mass fragmentation pattern of bithienylbenzamidine derivative **6**.

Structure of the newly synthesized bithiophene dicarbonitrile derivative **5** was assigned based on its elemental and spectral analyses. IR spectra of the bithiophene dinitrile **5** indicated the presence of nitrile group with stretching vibrations at 2212 cm^−1^. ^1^H-NMR spectrum of compound **5** displayed four doublet signals (one proton each) of bithiophene moiety at δ 7.57, 7.67, 7.89, 7.98 ppm, plus two signals integrated for three protons of 1,3,4-trisubstituted benzene moiety. Mass spectrum of compound **5** gave a molecular ion peak m/z at 310 (M^+^) as a base peak. The newly synthesized bithienylbenzamidine derivative **6** was assigned based on its elemental and spectral analyses. IR spectra of the diamidine **6** indicated the disappearance of the carbonitrile group and displayed new peaks corresponding for N–H stretching vibrations at ν′ 3342, 3187 cm^−1^. ^1^H-NMR spectrum of compound **6** gave four singlet signals at δ 9.22 (2H), 9.45 (2H), 9.51 (2H), 9.54 (2H) characteristic for the cationic diamidine groups, four doublet signals at δ 7.65 (1H), 7.66 (1H), 7.89 (1H), 8.13 (1H) of bithiophene-H′s, plus two signals integrated for three protons of 1,3,4-trisubstituted benzene moiety. ^19^F-NMR displayed a singlet signal at − δ112.76 ppm (using TFA as external standard) of fluorine atom. Mass spectrum of bithiophene diamidine compound **6** gave a m/z peak at 344 of its molecular ion peak (M^+^), and m/z peak at 327 (M^+^- NH_3_) as a base peak, along with a fragment with m/z peak at 310 due to loss of second molecule of ammonia (Fig. 3).

### Weight loss measurements

**Figure 4:**
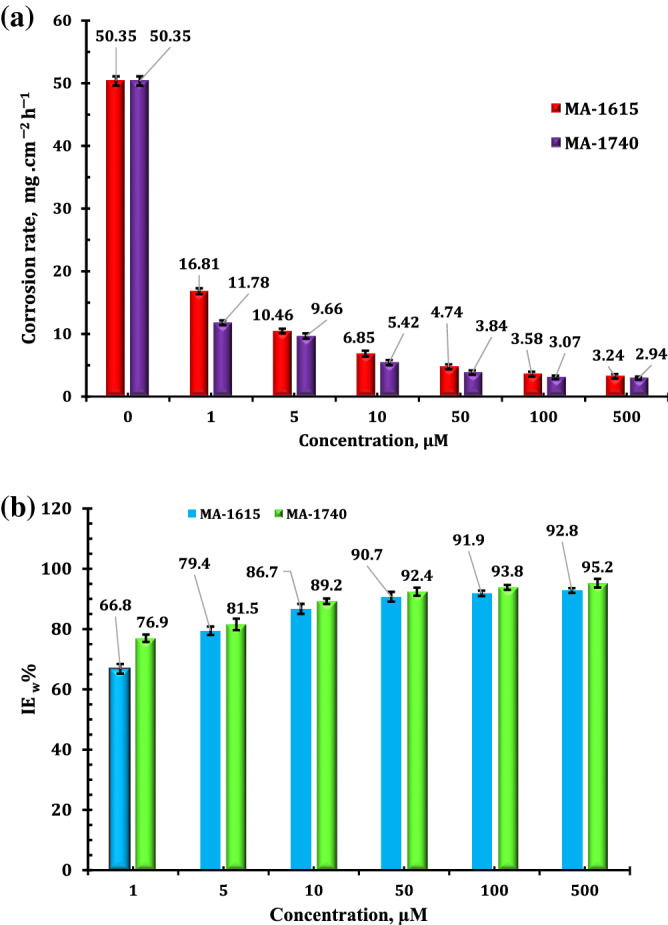
Variation of (**a**) corrosion rate and (**b**) inhibition efficiency with various doses of cationic aryl bithiophene/terthiophene additives by weight loss. Error bars (⊤) represent the standard deviations (%).

Weight loss is a generally used technique for estimating the corrosion inhibition efficiency of inhibitors, and it has high reliability because the average corrosion rate can be determined over time. The corrosion behavior of API 5L X70 steel in 1 M HCl without and with different doses of MA-1740 and MA-1615 was studied by weight loss method. Figure 4 shows the corrosion rate (k) and inhibition efficiency (IE_w_%) of the inhibitors. As shown in Fig. 4, the corrosion rate values are obviously reduced, and inhibition efficiency was increased after adding various doses of inhibitors. The maximum IE_w_% values of MA-1156 and MA-1740 are 93.57% and 94.16%, respectively. The inhibition behavior of the cationic aryl bithiophene/terthiophene derivatives can be explained by the adsorption of their molecules on the surface of carbon steel. According to Hammet substituent coefficient, MA-1740 has one more substituent –NH_2_ group that has negative value of σ (− 0.22) this indicates that capacity of MA-1740 to adsorb on metal surface is higher than MA-1615 as a result of increase in the electron density on the adsorption sites^26^.

### Electrochemical results

#### Potentiodynamic polarization (PP) studies

**Figure 5:**
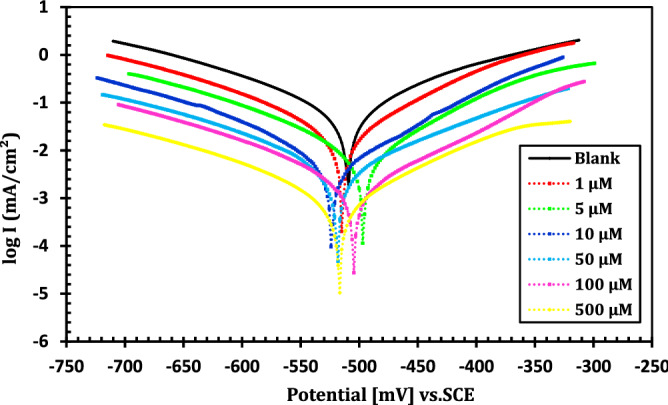
Potentiodynamic polarization curves of C-steel in 1 M HCl with different concentrations of inhibitor MA-1740 at 25 °C.

**Figure 6:**
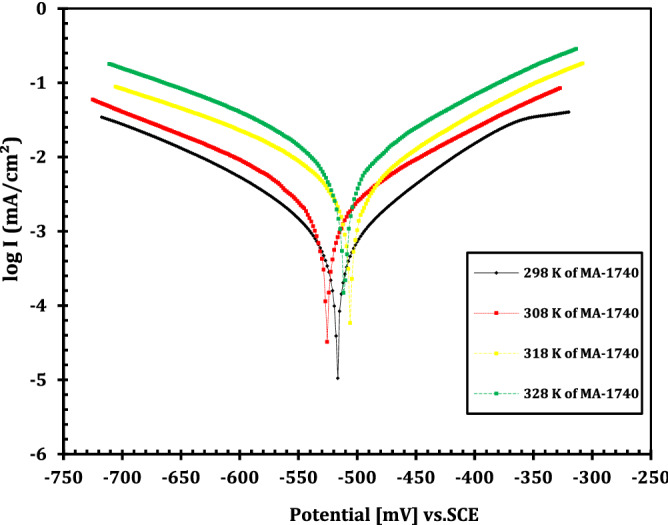
Effect of temperature on the Tafel polarization curves of C-steel in 1.0 M HCl with and without compounds.

Tafel plots of immersed API 5L X70 grade C-steel electrode in 1 M HCl without and with different doses of MA-1740 at 25 °C is represented in Fig. 5. Electrochemical variables obtained from extrapolation of Tafel curves were summarized in Table 3. It is obvious that with increasing the inhibitor concentration, the generated corrosion current density (*i*_corr_) for the API 5L X70 grade C-steel sample in the acidic medium shows a decreasing trend. This behaviour demonstrates the ability of the cationic aryl bithiophene/terthiophene derivatives to increase in the inhibition efficiencies and to reduce the corrosion rate caused via acidic solution. Looking at the PP plots showed that the anodic and cathodic lines, it is obvious that the curves tend to decline, which is an indication of the adsorption of cationic aryl bithiophene/terthiophene derivatives on the C-steel surface lower cathodic hydrogen evolution and anodic dissolution of Fe ions. Additionally, the difference in the values of Tafel slopes (*β*_a_ and *β*_c_) with different doses of cationic aryl bithiophene/terthiophene derivatives was small, indicating that the cationic aryl bithiophene/terthiophene derivatives hindered the corrosion of API 5L X70 type C-steel via inhibiting both anodic and cathodic reactions, without changing the corrosion process mechanism^27,28^. Tafel polarization was carried out at different temperature for the dicationic bithiophene inhibitor MA-1740 at optimum concentration of study as in Fig. 6. The corrosion measurements by Tafel polarization at different temperature are summarized in Table 4. From the results, it is obvious that the current density is higher when increasing the temperature, and so does the desorption rate of compounds on metal surface. However, this has no big shift in plots as can be seen in Fig. 6, this indicates no change in adsorption mechanism of compounds. The adsorption of cationic aryl bithiophene/terthiophene derivatives on API 5L X70 can be attributed to the interaction between the lone pairs of sulfur and nitrogen atoms present in these derivatives and the empty d-orbital of API 5L X70-type C-steel surface via coordination bonds. The resulting protection was achieved with the creation of a barrier film and led to inhibit corrosion. The inhibition efficiency (*η*_p_) can be calculated by the following equation^29,30^:

3 $${\eta }_{p}=\left[\frac{{i}_{corr}^{0}-{i}_{corr}}{{i}_{corr}^{0}}\right]\times 100,$$

where ${i}_{corr}^{0}$ and ${i}_{corr}$ are the corrosion current density without and with the studied inhibitors, respectively. From Table 3, the presence of two cationic aryl bithiophene derivative make the corrosion potential (*E*_corr_) to change to more positive and negative potentials compared with the blank solution. The *E*_corr_ values were significantly less than 85 mV/SCE, indicating that the studied cationic aryl bithiophene/terthiophene derivatives were mixed-type inhibitors^31–33^. In addition, the inhibitory activity of cationic aryl bithiophene/terthiophene improved by the presence of lone pairs of electrons and thiophene moiety in these compounds, as well as number of cationic amidine groups. Interestingly, addition of second cationic amidine group improvement outweighs the addition of third thiophene group and this may be attributed to solubility effect of the cationic group. For that reason, cationic aryl bithiophene/terthiophene derivatives can be ordered according to their efficiency as anti-corrosion of API 5L X70-type C-steel in HCl solution as follows: MA-1740 > MA-1615.

**Table 3:** Electrochemical kinetic parameters obtained from the polarization measurements on C-steel in uninhibited and inhibited acid solutions with different inhibitors doses at 25 °C.

Inhibitor	*C* (µM)	− *E*_corr_ (mV_SCE_)	*i*_corr_ (mA cm^−2^)	*β*_a_ (mV dec^−1^)	− *β*_c_ (mV dec^−1^)	*θ*	*η*_p_ (%)
Blank	–	509.2 ± 4	0.6381 ± 0.03	107.2 ± 1.4	149.5 ± 2.3	–	–
MA-1615	1	502.1 ± 6	0.1829 ± 0.06	85.3 ± 1.6	160.2 ± 1.9	0.7134	71.34
	5	511.5 ± 3	0.1501 ± 0.07	119.4 ± 1.9	134.1 ± 1.4	0.7648	76.48
	10	517.3 ± 5	0.1145 ± 0.08	98.3 ± 2.1	171.4 ± 1.7	0.8206	82.06
	50	506.9 ± 2	0.0924 ± 0.02	126.1 ± 1.3	153.7 ± 1.8	0.8552	85.52
	100	488.4 ± 7	0.0689 ± 0.05	87.9 ± 2.1	182.3 ± 0.9	0.8920	89.20
	500	493.7 ± 3	0.0278 ± 0.04	93.2 ± 1.6	131.6 ± 0.7	0.9564	95.64
MA-1740	1	514.9 ± 5	0.1578 ± 0.08	121.9 ± 1.3	138.2 ± 0.6	0.7527	75.27
	5	497.1 ± 4	0.1402 ± 0.05	106.1 ± 0.7	169.4 ± 2.4	0.7803	78.03
	10	524.2 ± 2	0.1036 ± 0.06	86.5 ± 1.1	218.1 ± 1.9	0.8376	83.76
	50	518.3 ± 6	0.0798 ± 0.07	114.3 ± 0.6	171.5 ± 0.8	0.8749	87.49
	100	504.7 ± 3	0.0554 ± 0.03	129.4 ± 1.5	164.9 ± 1.6	0.9132	91.32
	500	516.4 ± 8	0.0197 ± 0.02	76.2 ± 0.9	172.6 ± 0.7	0.9691	96.91

**Table 4:** Electrochemical kinetic parameters obtained from the polarization measurements on C-steel in uninhibited and inhibited acid solutions with optimum inhibitors doses at different temperature.

Inhibitor	T (K)	* − E*_corr._ mV(vs. SCE )	*I*_corr._, mAcm^−2^	*β*_a_ , mVdec^−1^	− *β*_c_, mVdec^−1^	*θ*	*IE* %
Blank	298	509.2 ± 4	0.6381 ± 0.03	107.2 ± 1.4	149.5 ± 2.3	–	–
	308	504.6 ± 3	0.9613 ± 0.06	94.1 ± 1.8	153.7 ± 1.8	–	–
	318	512.9 ± 2	1.2541 ± 0.08	91.6 ± 1.5	128.9 ± 0.7	–	–
	328	517.5 ± 6	1.8562 ± 0.02	87.3 ± 0.9	146.1 ± 2.1	–	–
MA-1615	298	493.7 ± 3	0.0278 ± 0.04	93.2 ± 1.6	131.6 ± 0.7	0.9564	95.64
	308	489.1 ± 5	0.1135 ± 0.05	101.8 ± 1.2	183.4 ± 1.5	0.8819	88.19
	318	497.3 ± 2	0.1943 ± 0.03	94.1 ± 0.8	176.9 ± 1.2	0.8451	84.51
	328	508.9 ± 4	0.3849 ± 0.07	89.3 ± 1.3	165.1 ± 0.9	0.7926	79.26
MA-1740	298	516.4 ± 8	0.0197 ± 0.02	76.2 ± 0.9	172.6 ± 0.7	0.9691	96.91
	308	525.6 ± 3	0.0891 ± 0.05	104.1 ± 0.6	159.3 ± 1.4	0.9073	90.73
	318	506.3 ± 5	0.1796 ± 0.04	96.2 ± 1.3	178.4 ± 1.2	0.8568	85.68
	328	511.6 ± 7	0.3454 ± 0.01	103.4 ± 1.7	185.1 ± 0.9	0.8139	81.39

#### Electrochemical impedance spectroscopy (EIS) test

**Figure 7:**
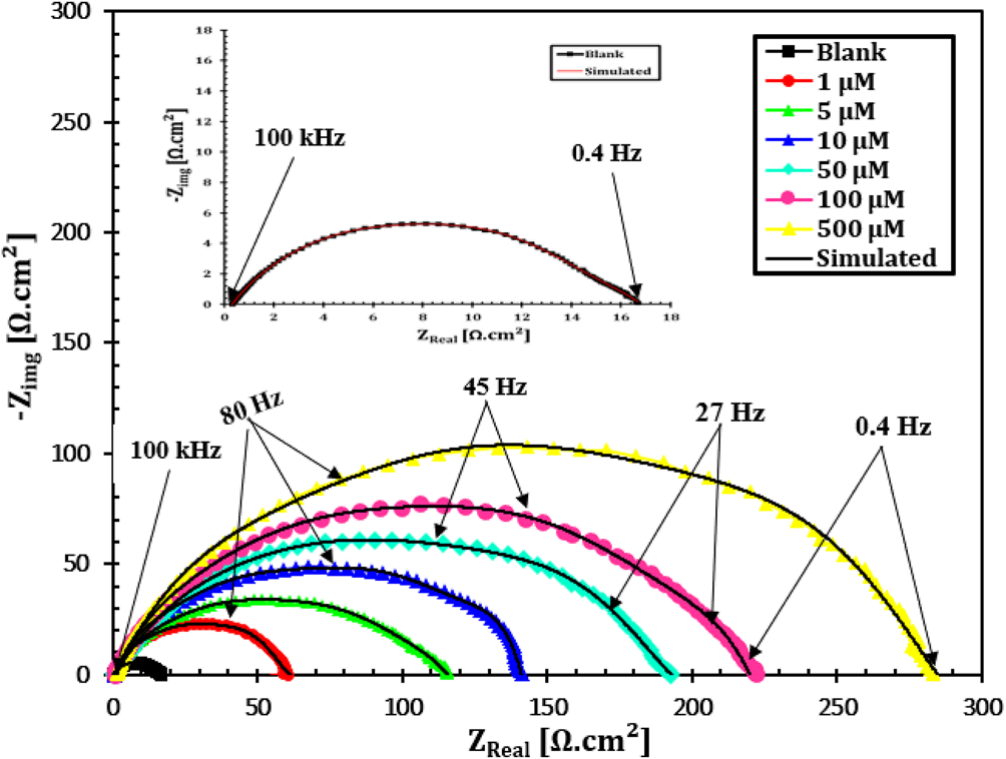
Nyquist plots of C-steel in 1 M HCl with different concentrations of inhibitor MA-1740 at 25 °C.

**Figure 8:**
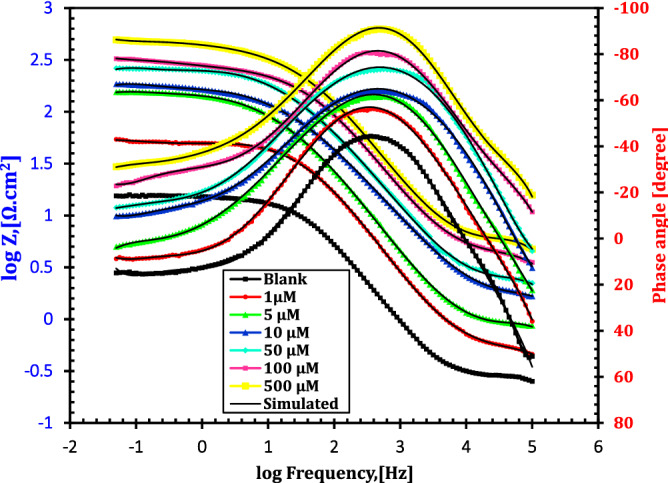
Bode plots of C-steel in 1 M HCl with different concentrations of inhibitor MA-1740 at 25 °C.

EIS is a widely utilized tool in corrosion inhibition studies of metals^34^. The corrosion behaviour of API 5L X70-type C-steel in HCl solution without and with different doses of the cationic aryl bithiophene/terthiophene derivatives was demonstrated by impedance tests. Figures 7 and 8 show the Nyquist and Bode plots, respectively of immersed API 5L X70 grade C-steel electrode in 1 M HCl solution without and with different doses of dicationic compound MA-1740 inhibitor at 25 °C. The electrochemical impedance parameters were summarized in Table 5. Nyquist plot in Fig. 7 displayed single capacitive loops, presenting that the dissolution process of API 5L X70 grade C-steel electrode in 1 M HCl solution in the absence and presence of inhibitors undergoes through charge-transfer and double-layer capacitance at the interface of C-steel/solution. As shown in Figs. 7 and 8, the Nyquist and Bode plots show the same style, inferring that the mechanism of cathodic and anodic corrosion reactions was not altered upon addition of cationic aryl bithiophene/terthiophene inhibitors. Whereas, the semicircles diameter becomes a little wider with raising the doses of studied cationic aryl bithiophene/terthiophene derivatives in 1 M HCl corrosive solution^35,36^. In addition, the Bode curves for the studied inhibitors in Fig. 8 exposed that the phase angle and the absolute impedance curves turned wider and larger with the rise in the inhibitor doses; such modifications support the inhibiting action of these inhibitor molecules against the corrosion of API 5L X70 type C-steel in 1 M HCl solution^37^.

**Table 5:** Electrochemical parameters obtained from EIS equivalent circuit fitting of the C-steel immersed in 1 M HCl without and with various doses of inhibitor.

Inhibitor	Conc. (µM)	*R*s (Ω cm^2^)	R^2^	*n*	*C*_dl,_ (µF/cm^2^)	*R*_ct,_ (Ω cm^2^)	*IE* %
Blank	–	1.391 ± 0.02	0.996	0.919 ± 0.03	107.23 ± 0.8	15.4 ± 0.5	–
MA-1615	1	2.723 ± 0.06	0.986	0.992 ± 0.04	51.36 ± 0.5	50.3 ± 1.3	69.5
	5	1.182 ± 0.12	0.964	0.994 ± 0.05	47.51 ± 1.2	58.7 ± 0.9	73.8
	10	1.305 ± 0.03	0.971	0.963 ± 0.02	36.57 ± 0.7	79.1 ± 0.6	80.6
	50	1.578 ± 0.12	0.966	0.999 ± 0.08	27.19 ± 0.9	94.5 ± 0.7	83.7
	100	1.239 ± 0.04	0.985	0.973 ± 0.01	20.31 ± 0.5	126.8 ± 1.4	87.9
	500	1.687 ± 0.05	0.982	0.964 ± 0.07	9.05 ± 0.3	224.2 ± 0.8	93.1
MA-1740	1	1.745 ± 0.43	0.993	0.959 ± 0.02	48.39 ± 1.1	57.3 ± 0.2	73.2
	5	1.953 ± 0.06	0.992	0.984 ± 0.06	42.16 ± 1.4	67.1 ± 0.6	77.1
	10	1.523 ± 0.39	0.979	0.995 ± 0.05	34.23 ± 0.7	82.4 ± 0.7	81.4
	50	1.314 ± 0.17	0.984	0.998 ± 0.03	23.15 ± 0.9	108.9 ± 1.1	85.9
	100	1.295 ± 0.23	0.997	0.995 ± 0.04	16.42 ± 0.6	143.5 ± 0.9	89.3
	500	1.791 ± 0.04	0.983	0.982 ± 0.07	5.24 ± 0.2	286.2 ± 1.3	94.6

**Figure 9:**
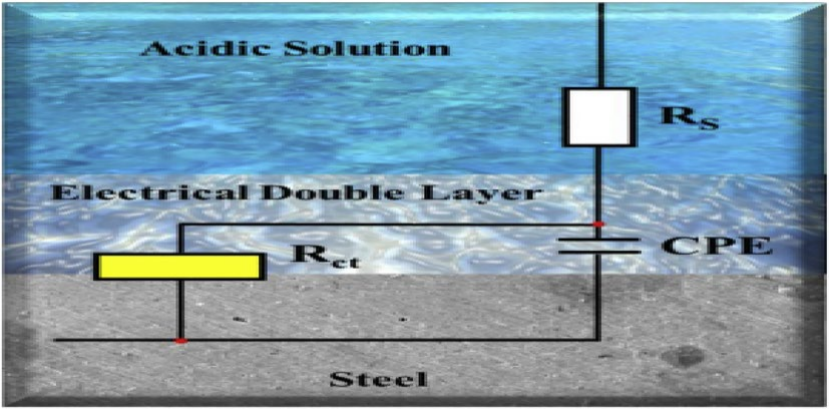
Equivalent circuit used to model metal/solution interface of C-steel in 1 M HCl without and with the cationic aryl bi-thiophene derivatives.

The equivalent circuit for data fitting experiments was presented in Fig. 9, and the fitted EIS data were given in Table 5. The electrical equivalent circuit includes solution resistance (*R*_s_), charge transfer resistance (*R*_ct_), and the constant phase element (*CPE*). *C*_dl_ is the double-layer capacitor. The *CPE* impedance (*Z*_CPE_) is calculated by the following equations^38–40^:

4 $${Z}_{CPE}=\frac{1}{{{Y}_{0}(j\omega )}^{n}},$$

5 $${C}_{dl}={{Y}_{0}({\omega }_{max})}^{n-1},$$

where *j* is the imaginary root (j^2^ = − 1), *Y*_0_ represents the modules of CPE, ω represents the angular frequency, *n* represents the deviation index in terms of a phase shift, 2 ${\omega }_{max}=2\pi {f}_{max}$, and *f*_max_ represents the frequency at the maximum value of the imaginary component of EIS spectra. The values of ${\eta }_{EIS}$ from EIS parameters of the cationic aryl bi-thiophene derivatives inhibitors can be obtained by the following equations^41^:

6 $${\eta }_{i}=\left[\frac{{R}_{ct}-{R}_{ct}^{0}}{{R}_{ct}}\right]\times 100,$$

where ${R}_{ct}$ and ${R}_{ct}^{0}$ denotes the charge transfer resistances for C-steel in inhibited and uninhibited acid test solutions, respectively. The data in Table 5 revealed that R_ct_ values is higher when the dose of cationic bithiophene/terthiophene additives increases in test solutions. While we notice drop in the values of C_dl_ with uplifting the concentration of inhibitors in acid medium. This action can be correlated to the adsorption of studied cationic bithiophene/terthiophene derivatives on C-steel/electrolyte interface that blocks corrosion reactions on C-steel surface and protect the metal from further acid attack^42^. The data in Table 5 revealed that the lower value of n in the blank solution (free from inhibitor molecules) can be attributed to the attack of aggressive ions on carbon steel surface leading to increase the heterogeneity of the surface. However, in the presence of inhibitor concentrations the values of n increase due to the formation of protection film and increasing the homogeneity of the surface^41,43^.

In view of the results presented in Table 5, the adsorption of the cationic bithiophene/terthiophene derivatives molecules onto the surface of API 5L X70 type C-steel occurs by substituting the existing water molecules and then creating a thicker and more ordered fencing protection layer. This has resulted in their ability to inhibit electron transfer and generally mitigate the corrosion reaction^44,45^. The EIS results revealed that the value of R_ct_ increases gradually with the increase in concentration of the synthesized inhibitors and this indicates an increase in the corrosion inhibition efficiency, which agrees with the potentiodynamic polarization results obtained.

Regression coefficients (R^2^) is listed in Table 5.

R^2^ is a statistic that will give some information about the goodness of fit of a model. In regression, the R^2^ coefficient of determination is a statistical measure of how well the regression predictions approximate the real data points. An R^2^ of 1 indicates that the regression predictions perfectly fit the data. The data in Table 5 revealed that R^2^ is an approximation of 1 indicating that the regression predictions fit well with the EIS data.

### Adsorption study

**Figure 10:**
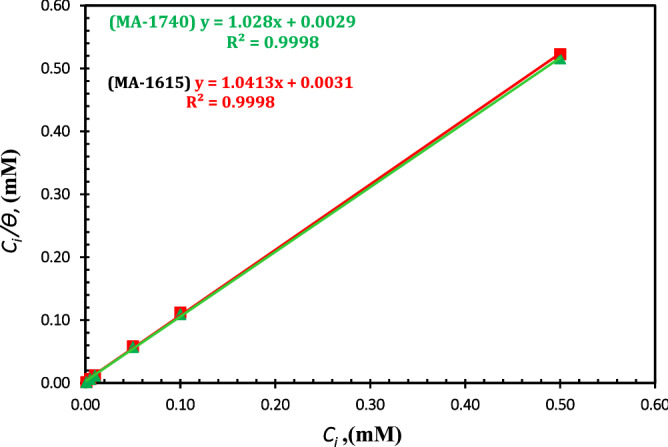
Langmuir adsorption plots for the cationic aryl bithiophene/terthiophene inhibitors on API 5L X70-type C-steel in 1 M HCl.

The inhibition potential of corrosion inhibitor depends on the adsorption behaviour on C-steel surface. To investigate the type adsorption reaction of the cationic aryl bithiophene/terthiophene derivatives on API 5L X70-type C-steel surface, the adsorption isotherms must be investigated. Langmuir adsorption isotherm showed the best fit for the tested cationic aryl bithiophene/terthiophene derivatives that is defined as follows^46^:

7 $$\frac{{C_{{}} }}{\theta } = \frac{1}{{K_{ads} }} + C,$$

where *K*_ads_ is the adsorption equilibrium constant, C represents the inhibitor concentration, and *θ* is the surface coverage. Figure 10 represents the plotted curves of *C*/*θ versus C* for the investigated compounds*,* which gave a straight line, and the extracted parameters were recorded in Table 6. The linear correlation coefficient (*R*^2^) and slope of these linear curves were found close to unity, indicating that the adsorption of these cationic aryl bithiophene/terthiophene derivatives on the surface of API 5L X70 type C-steel in 1 M HCl solution follow the Langmuir isotherm. The Gibbs standard free energy $(\Delta {G}_{ads}^{o})$, which is a useful thermodynamic parameter in our study was calculated as follows^18,47,48^:

8 $$\Delta {G}_{ads}^{o}=-RTln \left(55.5 {K}_{ads}\right),$$

where *R* is the gas constant, *T* represents the absolute temperature, and ${K}_{ads}$ is the adsorption equilibrium constant that can be calculated from the interception of the isotherm line with the C/θ axis. The values of *K*_ads_ and $\Delta {G}_{ads}^{o}$ were listed in Table 6. The higher values of *K*_ads_ indicate the strong adsorption ability of these studied cationic aryl bithiophene/terthiophene derivatives on the surface of API 5L X70 type C-steel in 1 M HCl solution, and then enhanced protection against corrosion. From the results in Table 6, the values of $\Delta {G}_{ads}^{o}$ are negative, which indicates that the adsorption of the studied inhibitors on C-steel surface is a spontaneous process. Inspecting the obtained values of $\Delta {G}_{ads}^{o}$ in Table 6, the Δ*G°*_ads_ values of the studied inhibitors ranged from − 33.88 to − 36.13 kJ mol^−1^, so it is the adsorption process occurs through both physical and chemical adsorption type of interactions^49–51^.

**Table 6:** Adsorption equilibrium constant (*K*_ads_) and standard free energy of adsorption (Δ*G°*_ads_) of the investigated inhibitors for C-steel in 1 M HCl solution at 25 °C.

Inhibitor	Slope	Regression coefficient (*R*^2^)	intercept	K_ads_ (L mol^–1^)	–Δ*G°*_ads_ (kJ mol^–1^)
MA-1615	1.0413	0.9998	0.0031	322,580.65	41.38
MA-1740	1.028	0.9998	0.0029	344,827.59	41.54

### UV–visible spectrometric measurements

**Figure 11:**
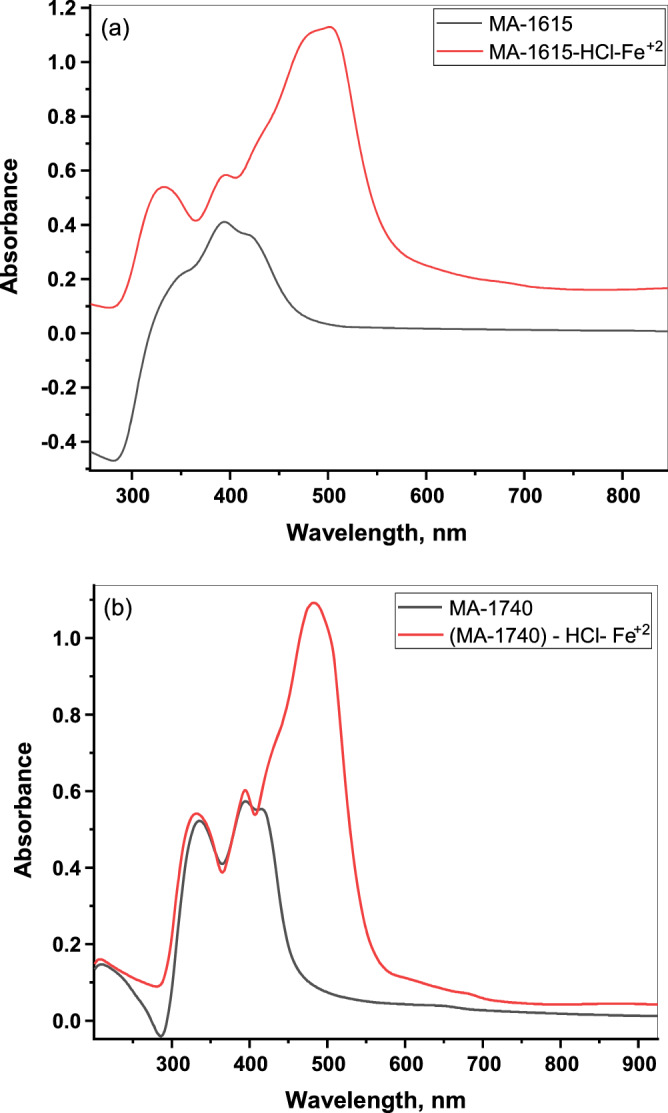
UV–visible electronic spectra of 500 µM of inhibitor MA-1615 (**a**) and inhibitor MA-1740 (**b**) before (black color) and (red color) after C-steel immersed in 500 µM HCl solution.

According to the literature reports, the influence of corrosion inhibition on metals in the test media in the presence of an inhibitor may be attributed to the complex formation between a metal and an inhibitor^52–54^. In our present work, in order to confirm the possibility of a complex formation between the C-steel electrode and the inhibitor molecules, UV–Vis spectrometric measurements were recorded for (500 µM) of the inhibitor, (500 µM) HCl solution containing (500 µM) of inhibitor after C-steel immersion for 28 h at 30 °C (Fig. 11a,b). The electronic spectra of investigated inhibitors (Fig. 11a,b) show visible peaks at (394, and 335 nm), and (396, and 337 nm) for MA-1615, and MA-1740, respectively. This may be attributed to the π–π* transition with an important charge transfer character. On the other hand, after the C-steel immersion in the test solution for 28 h at 30 °C (Fig. 11a,b) display new visible bands at (501, and 496 nm) for MA-1615, and MA-1740, respectively. This confirm the formation of a complex between the Fe^+2^ cations released during the corrosion reaction and the investigated inhibitors MA-1615, and MA-1740 molecules in (500 µM) HCl solution^55–57^.

### Atomic force microscopic (AFM) analysis

**Figure 12:**
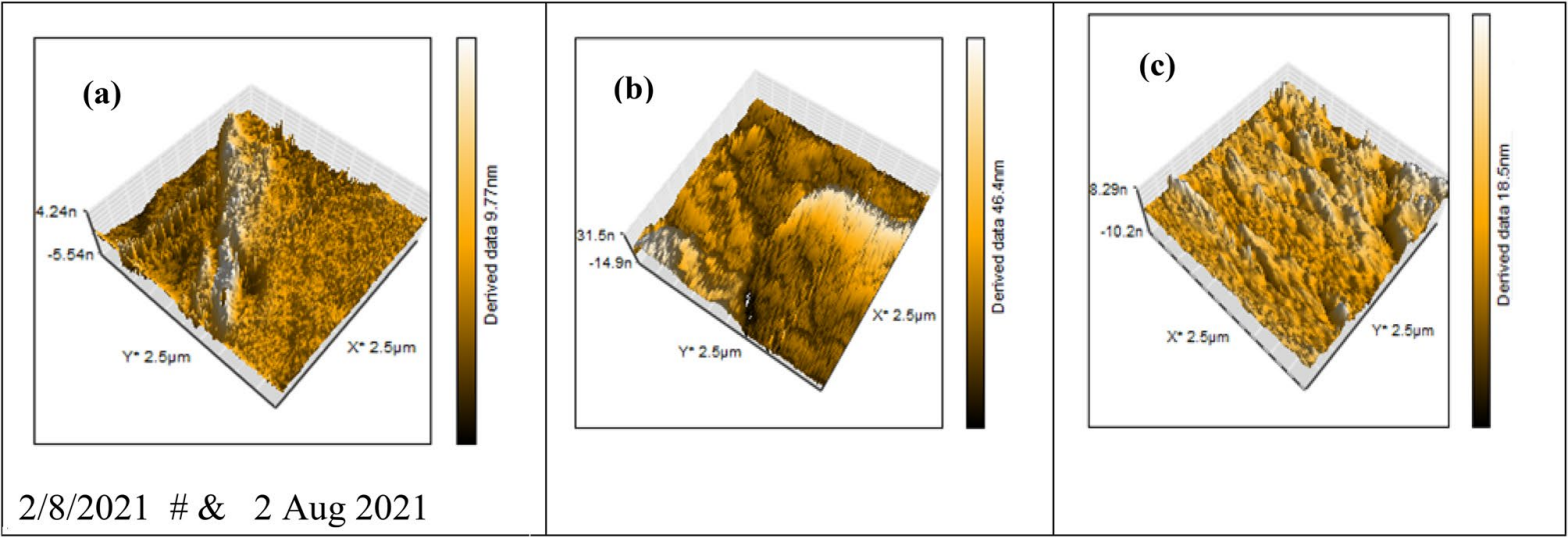
Three-dimensional AFM images for API 5L X70-type C-steel surface in 1 M HCl: (**a**) before immersion, **(b**) in 1 M HCl (Blank) and (**c**) in 1 M HCl containing 500 µM of inhibitor MA-1740.

Atomic force microscope (AFM) analysis is a powerful surface analysis technique to assess the morphological surface changes for the used substrate prior to and after the incorporation of corrosion inhibitors in test solutions. Figure 12a–c describes the AFM 3D images of polished C-steel surface and the surface of C-steel after 24 h immersion in blank 1 M HCl and another test solution with 0.01 M of inhibitor MA-1740 at room temperature. As shown in Fig. 12a, the API 5L X70 type C-steel before immersion was very smooth and homogeneous surface, while the surface of C-steel after immersion in 1 M HCl without inhibitor displayed a severely damaged and roughed surface as shown in Fig. 12b. Figure 12c depicts the 3D image of API 5L X70 type C-steel surface in 1 M HCl solution containing 0.001 M of inhibitor MA-1740 in which the surface has become flatter, and smoother compared to Fig. 12b. This is because the entire surface is insulated by a protective layer of inhibitor MA-1740. From the AFM analysis, the average surface roughness (*Ra*) obtained for the polished C-steel surface was 38.2 nm and the average surface roughness (*Ra*) of C-steel surface in 1 M HCl was 467.1 nm while the *Ra* value in the presence of 0.001 M of MA-1740 was 103.7 nm. These surface roughness values proved that the inhibitor MA-1740 molecules protect the surface of C-steel, most probably by forming a protective film from inhibitor molecules on API 5L X70 C-steel surface^58,59^.

### Corrosion inhibition mechanism

**Figure 13:**
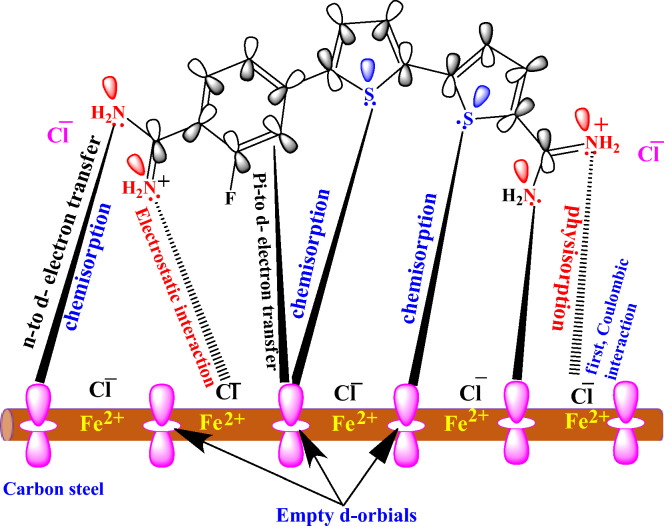
Possible adsorption mechanism of inhibitor MA-1740 on API 5L X70 type C-steel surface in HCl solution.

The cationic aryl bithiophene/terthiophene derivatives showed outstanding corrosion inhibitive properties which is attributed to the adsorption of these cationic aryl bithiophene/terthiophene derivatives on API 5L X70 type C-steel surface in 1 M HCl through either physisorption or chemisorption type of interaction as presented in Fig. 13. Thiophene rings and nitrogen atoms are considered the active binding sites in the adsorption mechanism. Physisorption involves first the columbic interaction among chloride ions, and then electrostatic interaction takes place between the positively charged N atoms in inhibitors and the negative charge on the chloride ions that adsorbed to the positively-charger API X70 type C-steel surface in acid solution. This interaction forms a thin barrier layer, inhibiting API X70 C-steel surface from reacting with corrosive species (physical adsorption). Moreover, the existence of chloride anions increases the capacity of adsorption compounds on API X70 C-steel surface^3^. The adsorption of these investigated compounds, on the other hand, can occur chemically onto the steel surface through the high electron density cloud of the π-electrons aromatic cycles that possess S heteroatoms, and the imine group (–C=N–) that facilitate the extension of the double bond conjugation on the whole structure enhancing the electron distribution and a more planar conformation on the substrate surface^60^. The chemical adsorption is also feasible via the coordinating bonds that can be formed between the lone-paint of electron on the heteroatoms (S) of thiophene rings and the vacant *d*-orbitals of Fe atoms in API 5L X70 type C-steel surface^61,62^. The variation between the two inhibitor molecules MA-1740, and MA-1615 in terms of their chemical structure feature was responsible for their different corrosion inhibition performance, as reported throughout all this study. Examining these structural deviations, the MA-1740 molecule possessed two amidine groups and two thiophene rings, and MA-1615 molecule possessed three thiophene rings and one amidine group. Accordingly, these cationic aryl bithiophene/terthiophene derivatives can build up a protective film on the surface of API 5L X70 type C-steel via physical and chemical reactions, protecting the X70 C-steel from further dissolution.

## Conclusion

1. Two novel cationic aryl bithiophene/terthiophene derivatives MA-1615, MA-1740 were synthesized, characterized by spectral means.
2. The new aryl bithiophene/terthiophene derivatives were investigated as corrosion inhibitors in HCl for API 5L X70 C-steel using electrochemical techniques, which showed higher corrosion inhibition action than similar compounds in structure in literature
3. The adsorption of the two investigated cationic inhibitors on API 5L X70 C-steel surface followed Langmuir isotherm and blocking the corrosion reaction sites. The negative sign of ${\Delta G}_{ads}^{^\circ }$ is an indication that the adsorption of cationic aryl bithiophene/terthiophene derivatives is a spontaneous process. ${\Delta G}_{ads}^{^\circ }$ values means that the adsorption involves both chemisorption and physisorption mechanisms.
4. Tafel curves indicated that the two investigated cationic inhibitors impede the cathodic and anodic reactions simultaneously, and therefore considered as mixed-type inhibitors. EIS measurements indicate that the increase in the inhibitor′s concentration is accompanied with a rise in the values of both *R*_ct_ and IE % and a decline in the value of *C*_dl_.
5. AFM pictures confirmed the presence of protective layer on API 5L X70 C-steel surface via the adsorption of the two investigated cationic bithiophene/terthiophene inhibitors. This protective layer protects API 5L X70 steel surface from further acid attack.
6. The presence of dicationic group in MA-1740 has more structural effect on increasing the corrosion inhibition than introducing third thiophene ring.

## Supplementary Information

Supplementary Information.
