# Supplementary material for: Novel cationic aryl bithiophene/terthiophene derivatives as corrosion inhibitors by chemical, electrochemical and surface investigations
Source: Sci Rep. 2022 Feb 24;12:3192. doi: 10.1038/s41598-022-06863-8 (PMC8873503; doi:10.1038/s41598-022-06863-8)
Supplement: Supplementary file 1 — Supplementary Information. [file 41598_2022_6863_MOESM1_ESM.docx]

**Novel cationic aryl bithiophene/terthiophene derivatives as corrosion inhibitors by chemical, electrochemical and surface investigations**

**Mohamed A. Ismail*^a^, Mahmoud M. Shaban ^b^, E Abdel-Latif ^a^, Fatma H. Abdelhamed^a^, Mohamed A. Migahed ^b^, Mahmoud N. El-Haddad ^a^, Ashraf S. Abousalem **^a, c^**

*^a^ Department of Chemistry, Faculty of Science, Mansoura University, Mansoura 35516, Egypt*

*^b^ Egyptian Petroleum Research Institute (EPRI), Nasr City 11727, Cairo, Egypt.*

*^c^ Quality Control Laboratory, Operation Department, JOTUN, Egypt*

______________________________

Correspondence to:

*Mohamed A. Ismail

Mansoura University, Faculty of Science, Department of Chemistry, Mansoura 35516, Egypt.

Email: [mismail@mans.edu.eg](mailto:mismail@mans.edu.eg)

**Ashraf S. Abousalem

Quality Control Laboratory, Operations Department, Jotun, Egypt.

E-mail : [ashraf.abousalem@gmail.com](mailto:ashraf.abousalem@gmail.com)

**I. Figures for NMR Spectra of the newly synthesized compounds**

**II. Figures for Mass Spectra of the newly synthesized compounds**

**I. Figures for NMR Spectra of the newly synthesized compounds**

| **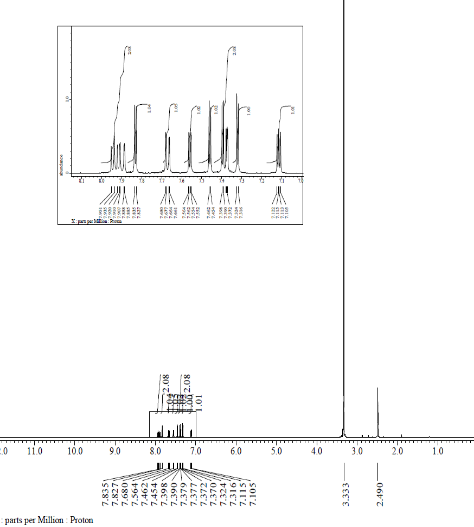**    **Compound 3:** ^1^H-NMR/JEOL 500 MHz |
| --- |

| **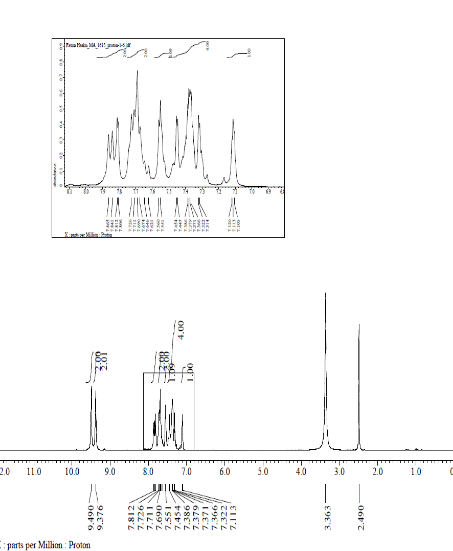**    **Compound 4:** ^1^H-NMR/JEOL 500 MHz |
| --- |

| **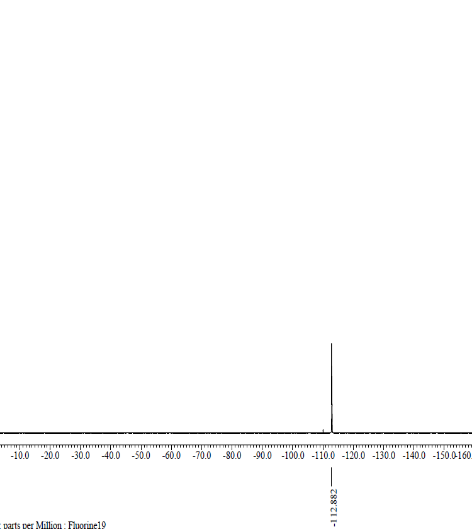**    **Compound 4:** ^19^F-NMR/JEOL 500 MHz |
| --- |

| **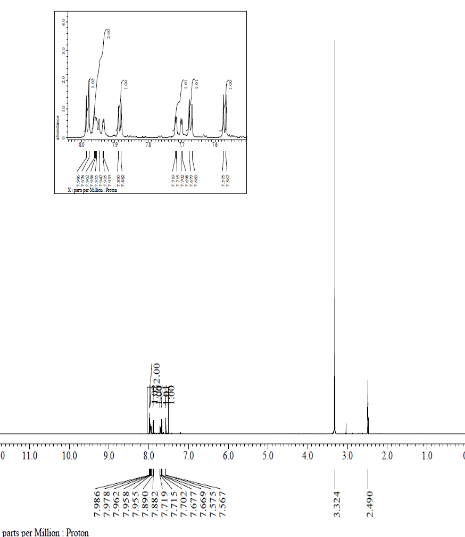**    **Compound 5:** ^1^H-NMR/JEOL 500 MHz |
| --- |

| **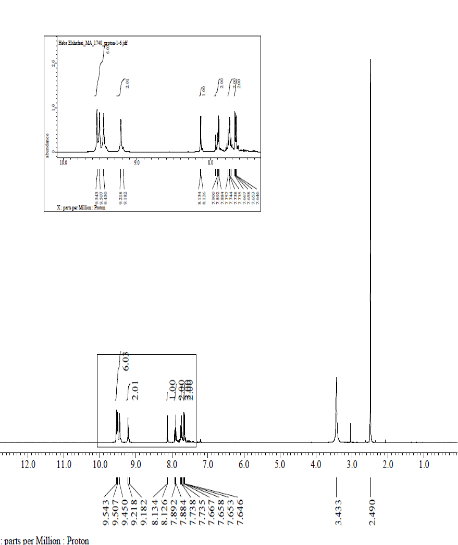**    **Compound 6:** ^1^H-NMR/JEOL 500 MHz |
| --- |

| **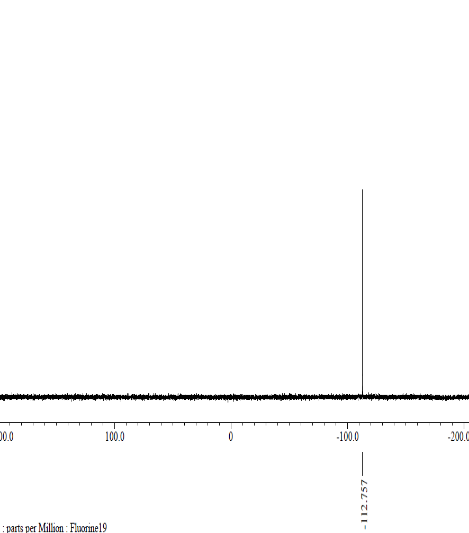**    **Compound 6:** ^19^F-NMR/JEOL 500 MHz |
| --- |

**II. Figures for Mass Spectra of the newly synthesized compounds**

| **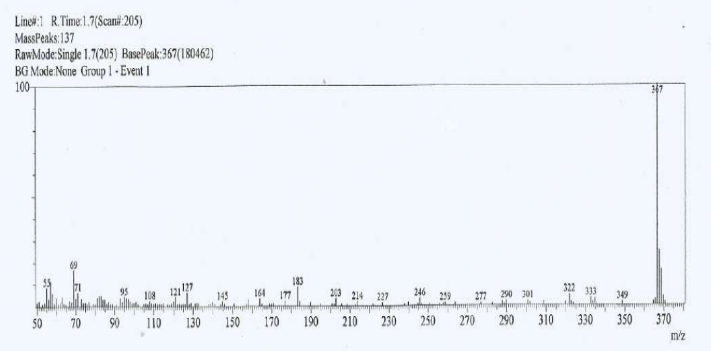**    **Compound 3: Mass Spectrum** |
| --- |

| **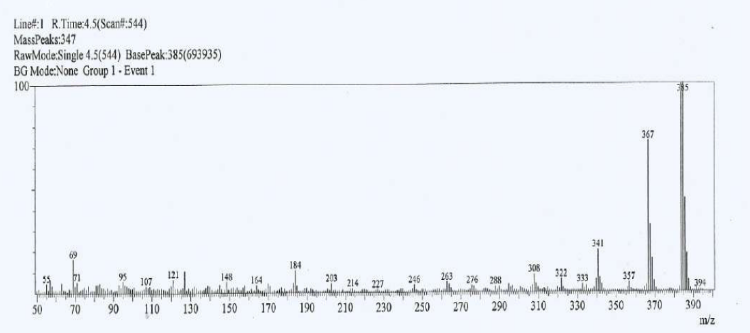**    **Compound 4 (free base): Mass Spectrum** |
| --- |

| **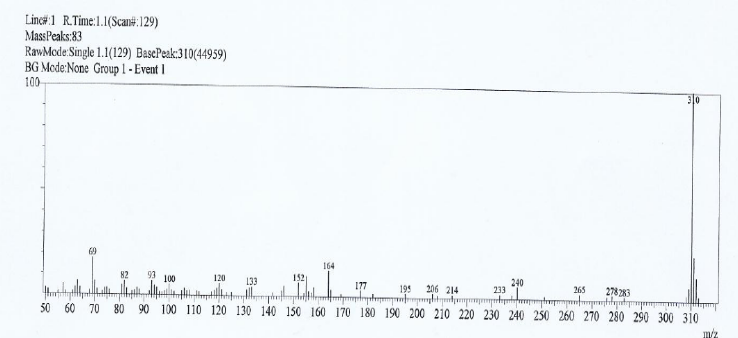**    **Compound 5: Mass Spectrum** |
| --- |

| **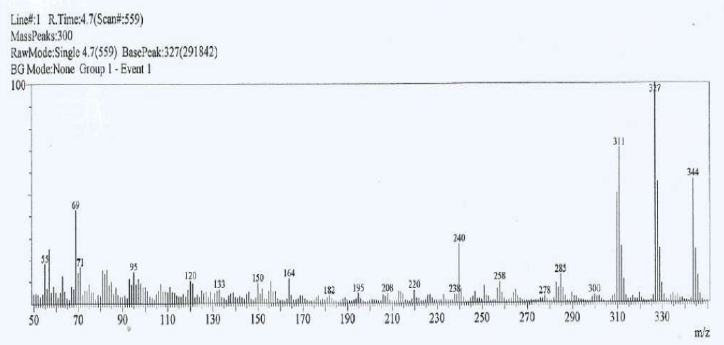**    **Compound 6 (free base): Mass Spectrum** |
| --- |
